# Author’s response to letter to editor for “Assessing the use of the novel tool Claude 3 in comparison to ChatGPT 4.0 as an artificial intelligence tool in the diagnosis and therapy of primary head and neck cancer cases”

**DOI:** 10.1007/s00405-026-10020-6

**Published:** 2026-03-05

**Authors:** Benedikt Schmidl, Tobias Hütten, Steffi Pigorsch, Fabian Stögbauer, Cosima C. Hoch, Timon Hussain, Barbara Wollenberg, Markus Wirth

**Affiliations:** 1 Department of Otolaryngology Head and Neck Surgery, Technical University Munich, Munich, Germany; 2 Department of Otorhinolaryngology, Medical University of Graz, Graz, Austria; 3 Department of RadioOncology, Technical University Munich, Munich, Germany; 4 Institute of Pathology, Technical University Munich, Munich, Germany; 5 Department of Otolaryngology Head and Neck Surgery, RWTH Aachen, Aachen, Germany

## Abstract

Claude 3 Opus

HNSCC

Multidisciplinary Tumorboard

Artificial Intelligence

LLM

We sincerely thank the authors for their friendly and constructive comment on our study, “Assessing the use of the novel tool Claude 3 in comparison to ChatGPT 4.0 as an artificial intelligence tool in the diagnosis and therapy of primary head and neck cancer cases”[1]. Their comment provides an accurate reflection of our results and raises points that further clarify the context and implications of our findings.

The authors emphasize the central role of diagnostic accuracy in the treatment of HNSCC. In our study, differences were observed between the two models for selected diagnostic components when assessed using the modified Artificial Intelligence Performance Instrument (AIPI). However, as highlighted in the Letter, these differences should be interpreted strictly as relative performance within an exploratory evaluation framework. This is especially important since our analysis also revealed clinically questionable outputs, such as frequent recommendations for EBV or HPV testing outside routine indications, which illustrates that even well-structured responses may not align with established clinical practice, reinforcing the need for expert interpretation. The Letter therefore reinforces that our findings should be viewed as hypothesis-generating rather than indicative of clinical readiness. As emphasized by the authors, any comparative language used in the original article refers solely to relative performance within the applied evaluation framework.

We fully agree with the authors’ comments on transparency and source verification of current LLMs. In our study, Claude 3 Opus did not provide citations, whereas ChatGPT 4.0 occasionally referenced guidelines or clinical studies. In MDT contexts, the absence of clearly traceable sources increases the risk that AI-generated suggestions may be interpreted as evidence-based despite uncertain provenance. Even when references are provided, they require independent verification. AI-generated recommendations therefore should be interpreted as provisional and illustrative rather than directive. Readers should therefore account for these risks by critically evaluating the reasoning structure in conjunction with established guidelines and expert clinical judgment.

With regard to therapy recommendations, both models generally proposed treatment options that were in line with the MDT’s modality spectrum, but important limitations were observed. Differences in suggested options, including occasional recommendations of primary immunotherapy without consideration of biomarker status, illustrate that AI-generated outputs may not reliably reflect guideline-based decision pathways. These observations should not be interpreted as indicating greater clinical appropriateness of one model over another. Rather, they highlight the limited capacity of current large language models to contextualize therapeutic recommendations beyond the information explicitly provided in the prompt. This reflects a deliberate aspect of our study design: we used a standardized MDT-style case vignette to allow comparability across cases, but this necessarily excludes many factors that influence real-world decision-making, such as detailed imaging findings, resectability, functional outcomes, patient preferences, and institutional practice patterns. Readers should therefore interpret therapy-related outputs as illustrative rather than patient-specific, and future studies should focus on whether expanded and differently structured clinical input improves contextual reliability rather than merely increasing the number or apparent sophistication of recommendations.

Finally, we share the concerns regarding data dependence and potential bias in large language models. In addition to limitations related to training data, our results demonstrate that prompt formulation itself can influence model output. We tested multiple prompt designs and selected the best-performing format, showing that relatively small changes in case presentation can alter recommendations. This further supports the need for standardized evaluation approaches and validation across different institutions and patient populations.

Beyond the specific findings of our study, the exchange with the authors highlights a broader issue in the evaluation of large language models in head and neck oncology. Comparisons based on relative performance metrics, while informative, are insufficient to assess clinical relevance. Greater emphasis is needed on interpretability, reproducibility, and contextual reliability, particularly in MDT environments. Addressing these issues will be essential for developing evaluation approaches that align with the realities of multidisciplinary decision-making and patient safety.

In summary, the authors’ comments have helped refine the interpretation of our study. The goal of our study was the exploratory assessment of large language models under defined conditions. At present, general-purpose LLMs may assist with information structuring or preliminary consideration of diagnostic and therapeutic options in head and neck cancer, but limitations in transparency, reproducibility, and individualization preclude their use beyond a supportive role within expert-led MDT decision-making.

## References

[1] Schmidl B et al (2024) Assessing the use of the novel tool Claude 3 in comparison to ChatGPT 4.0 as an artificial intelligence tool in the diagnosis and therapy of primary head and neck cancer cases. Eur Arch Otorhinolaryngol 39112556 10.1007/s00405-024-08828-1 PMC11512878

